# Meat consumption and risk of esophageal and gastric cancer in the Golestan Cohort Study, Iran

**DOI:** 10.1002/ijc.34056

**Published:** 2022-05-17

**Authors:** Giulia Collatuzzo, Arash Etemadi, Masoud Sotoudeh, Arash Nikmanesh, Hossein Poustchi, Masoud Khoshnia, Akram Pourshams, Maryam Hashemian, Gholamreza Roshandel, Sanford M. Dawsey, Christian C. Abnet, Farin Kamangar, Paul Brennan, Paolo Boffetta, Reza Malekzadeh

**Affiliations:** ^1^ Department of Medical and Surgical Sciences University of Bologna Bologna Italy; ^2^ Metabolic Epidemiology Branch, Division of Cancer Epidemiology and Genetics National Cancer Institute Bethesda Maryland USA; ^3^ Digestive Oncology Research Center, Digestive Diseases Research Institute Tehran University of Medical Sciences, Shariati Hospital Tehran Iran; ^4^ Golestan Research Center of Gastroenterology and Hepatology Golestan University of Medical Sciences Gorgan Iran; ^5^ Department of Biology, School of Art and Sciences Utica College Utica New York USA; ^6^ Department of Biology, School of Computer, Mathematical, and Natural Sciences Morgan State University Baltimore Maryland USA; ^7^ Section of Genetics International Agency for Research on Cancer Lyon France; ^8^ Stony Brook Cancer Center Stony Brook University Stony Brook New York USA

**Keywords:** esophageal cancer, gastric cancer, meat intake, processed meat, red meat, risk factor

## Abstract

Red meat and processed meat are associated with some gastrointestinal cancers. Our study aims to investigate the association of different meat types with esophageal and gastric cancer (EC, GC) in a high‐risk population. The Golestan Cohort Study (GCS) is a population‐based cohort of 50 045 individuals aged 40 to 75 from northeast Iran. Detailed data on different exposures were collected using validated questionnaires. We considered quintiles of meat consumption, using grams and density (g/1000 kcal/day). We calculated intake of red, processed, organ and white meat, as well as total red meat, including the first three. We used proportional hazards regression models to estimate hazard ratios (HRs) and corresponding 95% confidence intervals (CIs) for the association between meat types and cancer. During 12 years of follow‐up, out of 49 585 participants (57.4% women), 369 developed EC (48.2% women) and 368 developed GC (27.5% women), including 309 esophageal squamous cell, 20 esophageal adenocarcinomas, 216 cardia and 95 non‐cardia GC. No association was found for EC except for red meat among females (HR for one quintile increase 1.13, 95% CI = 1.00‐1.27). The risk of GC increased for intake of total red meat (HR 1.08, 95% CI = 1.00‐1.17) and red meat separately (HR 1.09, 95% CI = 1.00‐1.18). The HR for red meat and non‐cardia GC was 1.23 (95% CI = 1.02‐1.48). No associations were observed for other types of meat. In conclusion, in this high‐risk population red meat intake is associated with GC, but not EC, suggesting a substantial role of this modifiable factor in determining the burden of GC.

## Graphical abstract

What's new?

While high intakes of red meat and processed meat are risk factors for certain cancer types, especially colorectal cancer, associations with esophageal cancer (EC) and gastric cancer (GC) remain uncertain. Here, meat intake and EC and GC risk were examined in a population in northeast Iran with overall low meat intake, except for high consumption of organ and chicken meat. Hazards regression modeling reveals associations between red meat intake and elevated GC risk, particularly non‐cardia GC. There was no association with EC or its subtypes. Further study is needed to determine possible etiological involvement of red meat in GC development.

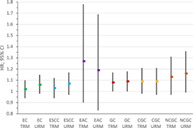

**Abbreviations**
BMI: body max index
CI: confidence interval
EAC: esophageal adenocarcinoma
EC: esophageal cancer
ESCC: esophageal squamous cell carcinoma
FFQ: food frequency questionnaire
GC: gastric cancer
GCS: Golestan Cohort Study
Hp: *Helicobacter pylori*
HR: hazard ratio
ICD: International Classification of Disease
NOC: *N*‐nitroso compound

## 1 INTRODUCTION

High intake of red meat and processed meat represents a potential risk factor for several types of cancer including in particular colorectal cancer, while an association has been suggested for esophageal cancer (EC) and gastric cancer (GC).^1^ Previous reviews have suggested a positive association between red meat and cardia GC, processed meat and non‐cardia GC among men and between both types of meat and esophageal squamous cell carcinoma (ESCC) among men.^2^, ^3^ These associations appear to be stronger in case‐control studies and less convincing in cohort studies.^1^ In addition, white meat intake has been inversely related to EC and GC in some studies.^4^, ^5^

The incidence of EC and GC is particularly high in Northern Iran,^6^, ^7^, ^8^, ^9^ and the burden of both diseases is predicted to increase by 2025 due to the increase in size and the aging of the population.^10^ The incidence of GC is decreasing in most countries, and this trend concerns mainly non‐cardia GC.^11^, ^12^ The main risk factors for EC vary by histological subtype, with cigarette smoking, alcohol drinking, mechanical or high‐temperature damage (eg, caustics, hot beverages) being the main risk factors of esophageal squamous cell carcinoma (ESCC), and obesity and gastroesophageal reflux disease those of esophageal adenocarcinoma (EAC).^13^ Infection with *Helicobacter pylori* (Hp) represents the main risk factor for non‐cardia GC, and obesity that of cardia GC.^14^ Thus, the investigation of environmental and lifestyle, including dietary risk factors among the Iranian population raises particular interest with the aim of explaining the high incidence of these cancers and the peculiar pattern of their subtypes.^15^ Due to cultural reasons, Golestan Province in Iran is a low meat‐intake population, with a relatively high share of organ and chicken meat, especially in rural areas.

We aimed at exploring the association between meat intake and both EC and GC, stratifying by cancer subtype and type of meat, in a large prospective cohort study from Northern Iran. Investigating this link in a high‐risk population with low meat consumption raises valuable insights into the possible causal relationship, contributing to the etiologic knowledge of EC and GC.

## 2 METHODS

The Golestan Cohort Study (GCS) is a prospective population‐based cohort study including 50 045 participants (21 234 men and 28 811 women) aged 40 to 75 years at baseline, which was set up in January 2004 in northeastern Iran (Golestan Province) to study risk factors of EC and GC in this high‐risk population. Participants were selected by random sampling of subjects without a history of EC or GC living in Gonbad City and 326 villages in the Province. Trained interviewers used a general questionnaire to collect data at baseline on demographic characteristics, residential history, occupation, education, physical activity, medical history and lifestyle habits including opium use, tobacco use, tea temperature and alcohol drinking. Height, weight and waist and hip circumferences were also measured. We calculated body mass index (BMI) by dividing weight (in kg) by height square (in m^2^). Nutritional data were collected at baseline using an extensive food frequency questionnaire (FFQ) that had been previously designed and adapted for the local context and successfully validated.^16^ Participants were asked about their frequency of consumption of a given serving of each food item daily, weekly or monthly during the year leading up to their recruitment. The daily intake of each food item was calculated by multiplying the frequency of consumption by the typical portion size. Then daily intake was converted into grams (g).

Meat items listed on the FFQ included red meat, processed meat, organ meat, white meat, including chicken (see Refs. [16, 17] for details). Different types of meat were considered and combined in variables used for the analysis, including unprocessed red meat (lamb, beef and hamburger), organ meat (eg, liver, kidney and heart), processed meat (sausage and deli meat) and white meat (chicken and fish, of which chicken represented the large majority). A term for total red meat intake was created including unprocessed and processed red meat, as well as organ meat. We calculated the density of meat consumed, by dividing the amount of meat by total caloric intake (g/1000 kcal/day).^18^ Subjects were also asked how they were used to prepare their food (shallow‐ or deep‐frying, barbecuing, boiling, steaming or other), separately for meat and fish.

Since enrolment, all GCS participants have been actively followed‐up for end‐points by annual telephone surveys and home visits. From January 2004 to December 2020, after a mean follow‐up time of 12.3 years, 49 528 subjects (99.0%) have been successfully monitored. Cases of cancer were identified during the active follow‐up, and by matching cohort members to the Golestan Population‐based Cancer Registry database.^10^ For those subjects who were reported dead, a verbal autopsy was performed, to identify specific causes of death.^19^ The total number of deaths during the follow‐up was 8727 (17.4% of cohort members). EC and GC were the two main causes of cancer deaths (1651 cancer deaths, of whom 304 from EC and 324 from GC). Cancer cases and deaths were coded according to the 10th Edition of the International Classification of Diseases (ICD10).

In this analysis, we considered cases or deaths from EC (ICD10 code C15) and GC (ICD10 code C16). Person‐years for EC and GC were calculated from the date of enrolment to 31 December 2020, date of diagnosis or death from EC or GC or last date of follow‐up, whichever came first.

Further details on the GCS have been described elsewhere.^20^ We excluded the individuals who were lost to follow‐up (N = 517), as well as the subjects corresponding to the extreme values for BMI (<16 and >45 kg/m^2^). Similarly, subjects with extreme values (highest 1% and lower 1% of the distribution) of sex‐specific total caloric intake were excluded. After these exclusions, data from 49 585 participants, including 21 101 men (42.6%) and 28 484 women (57.4%), with mean age at enrolment 52.06 (95% CI = 51.98‐52.14) were available for analysis.

For the purpose of this analysis, participants were divided into five quintiles according to intake of each type of meat. People who did not eat any type of meat were included in the lowest quintile. The outcomes included in this analysis, combining incident cases and deaths, were EC, distinguished in esophageal squamous cell carcinoma (ESCC) and esophageal adenocarcinoma (EA) when histological classification was available, and GC, including cardia GC (ICD‐10 code C16.0) and non‐cardia GC (ICD 10 codes C16.1‐C16.6).

Cox proportional hazards models were used to estimate unadjusted and adjusted hazard ratios (HRs) and 95% confidence intervals (CIs) for risk of EC, GC and cancer subtypes‐specific in each quintile of meat consumption, using the lowest quintile as reference category. We used both the addition and the substitution method of analysis,^21^ in order to disentangle the effect of the different types of meat. In particular, in the addition method the variables for each type of meat were included in the same models, while in the substitution method each meat type was included individually together with a variable for overall meat intake. We present as main results those based on the addition method.

In order to exclude the role of potential confounding by other factors, all multivariate models were adjusted for age, sex, BMI, ethnicity and place of residence, and selected additional variables were identified through backward selection. In particular, we kept those variables whose removal resulted in a 10% or larger change in the HRs for the main exposure variables. This resulted in different models chosen for each outcome. The covariates considered for additional adjustment included education, cigarette smoking, opium use, fruit consumption, vegetable consumption, salt intake and hot tea consumption. The final model for GC included education and fruit as additional covariates, while that for EC included education and hot tea consumption. Alcohol drinking was excluded because of the very small consumption (about 1% of the study population being regular alcohol drinkers). Details on the covariates and the models for cancer subtypes are shown in Table S1.

Additionally, we investigated whether the different types of cooking (shallow‐ and deep‐frying, barbecuing, boiling, steaming or other) modified the observed associations. We stratified the analysis by sex, Turkmen ethnicity and opium use, the latter being a major risk factor of upper‐GI cancer in this population.^22^ We assessed the heterogeneity across strata using the *Q* statistics.

For dose‐response analyses, we calculated the HR and 95% CI for the increase in one quintile of each variable of meat intake, together with tests for linear trend across quintiles. *P*‐values less than .05 were considered statistically significant. We calculated the proportion of cases attributable to consumption of meat above the first quintile according to the formula by Hanley.^23^

The package STATA V. 16 was used for all analyses.^24^

## 3 RESULTS

Our study population included 42.6% men (mean age 52.7, mean BMI = 25.1) and 57.4% women (mean age 51.5, mean BMI = 27.8). A total of 10.8% of subjects were current smokers, 14.7% were current opium users, 74.4% were of Turkmen ethnicity, 70.1% had less than 5 years of education and 79.9% were from the rural areas. Further details on sociodemographic characteristics of the study population according to red meat consumption are provided in Table 1.

**TABLE 1:** Selected characteristics of the study population by quintiles of total red meat intake (density method)

	Q1 (n = 9422)	Q2 (n = 9652)	Q3 (n = 9899)	Q4 (n = 9529)	Q5 (n = 9598)
Mean intake of total red meat (g/day)	2.82	7.99	13.7	21.5	42.8
Mean density intake of total red meat (g/1000 kcal/day)	1.36	3.74	6.28	9.77	19.4
Mean intake of white meat (g/day)	75.7	69.9	67.1	66.0	59.8
Mean calorie intake (kcal/day)	2013.2	2133.8	2182.6	2197.5	2205.7
Mean age	53.3	52.2	51.9	51.5	51.3
Sex (% female)	61.1	59.6	58.0	56.1	53.2
Mean BMI	26.1	26.5	26.8	26.9	27.0
Turkmen ethnicity (%)	59.2	74.9	77.4	79.1	78.8
<5 year education (%)	78.4	72.9	68.8	66.4	63.6
Physical activity (% lower tertile)	36.1	34.2	33.9	34.6	33.6
Current smokers (%)	8.34	9.80	10.4	11.0	14.2
Current opium users (%)	15.4	14.4	14.2	14.1	15.0
Hot tea drinkers (%)	59.9	59.8	61.2	61.8	62.1
Mean fruit intake (g/day)	106.8	133.5	152.3	167.2	189.3
Mean vegetable intake (g/day)	166.2	177.7	185.6	190.2	197.8
N EC cases	67	71	72	77	68
N GC cases	54	69	84	78	67

The cohort reported a mean intake of 18.4 g/day (95% CI = 17.8‐18.6) for total red meat, and 72.1 g/day (95% CI = 70.3‐73.8) for white meat. These values correspond to an intake density of 8.11 g per 1000 kcal/day (95% CI = 8.05‐8.19) and 32.3 g (95% CI = 32.0‐32.5) per 1000 kcal/day for total red and white meat, respectively.

The number of EC cases or deaths occurred in the cohort was 369, including 309 ESCC and 20 EAC cases (information on histology was missing for 40 cases). Cases of GC were 368, of which 216 cardia and 97 non‐cardia (information on topography was missing for 55 cases). Details on meat intake for EC and GC case status are shown in Table 2.

**TABLE 2:** Selected characteristics of the total cohort, as well as esophageal cancer (EC) and gastric cancer (GC) cases

	Total cohort	EC cases	GC cases
Mean total red meat (grams; density^a^)	18.2 g, 8.12	16.9 g, 7.92	19.7 g, 8.55
Mean white meat (grams; density^a^)	72.1 g, 32.3	67.6 g, 31.4	67.4 g, 30.4
Mean unprocessed red meat (grams; density^a^)	13.0 g, 5.77	12.7 g, 5.99	14.5 g, 6.35
Mean organ meat (grams; density^a^)	3.27 g, 1.5	2.74 g, 1.22	3.22 g, 1.38
Mean processed meat (grams; density^a^)	1.93 g, 0.85	1.54 g, 0.70	1.97 g, 0.82
Mean age (years)	52.0	58.8	58.6
Sex (% female)	57.4%	48.2%	27.5%
Mean BMI (kg/m^2^)	26.7	24.2	25.5
Turkmen ethnicity (%)	74.5%	86.5%	79.9%
<5 year education (%)	70.1%	84.8%	80.4%
Low physical activity (% lower tertile)	34.5%	45.7%	39.8%
Current smokers (%)	10.8%	14.6%	16.3%
Current opium user (%)	14,7%	22.8%	22.0%
Hot tea drinkers (%)	60.7%	68.3%	68.8%
Mean fruit intake (g/day)	151.47 g	140.6 g	150.0 g
Mean vegetable intake (g/day)	184.3 g	171.1 g	178.1 g

^a^ Grams/1000 kcal.

Tables 3 and 4 illustrate the HRs for EC and GC by quintiles of meat consumption based on the addition method. Corresponding results for cancer subtypes are shown in Figure S1.

**TABLE 3:** Hazard ratios of esophageal cancer for quintiles of meat consumption

N case/non‐case of esophageal cancer	Q1	Q2	Q3	Q4	Q5	
	68/9438	73/9689	72/9920	78/9539	70/9612	
	HR (95% CI)	HR (95% CI)	HR (95% CI)	HR (95% CI)	HR (95% CI)	Continuous^a^
Total red meat	1.00 (Ref)	1.02 (0.72‐1.43)	1.02 (0.72‐1.44)	1.15 (0.82‐1.61)	1.04 (0.73‐1.49)	1.02 (0.94‐1.10)
Red meat	1.00 (Ref)	0.99 (0.69‐1.43)	1.17 (0.82‐1.67)	0.97 (0.66‐1.43)	1.34 (0.93‐1.93)	1.06 (0.98‐1.15)
Processed meat	1.00 (Ref)	0.92 (0.64‐1.32)	1.15 (0.84‐1.58)	0.88 (0.61‐1.28)	0.87 (0.59‐1.27)	0.97 (0.90‐1.05)
Organ meat	1.00 (Ref)	1.20 (0.79‐1.80)	0.98 (0.64‐1.51)	1.10 (0.72‐1.67)	0.94 (0.61‐1.45)	0.96 (0.88‐1.04)
White meat^b^	1.00 (Ref)	0.85 (0.62‐1.17)	0.59 (0.41‐0.85)	0.83 (0.60‐1.16)	0.85 (0.61‐1.18)	0.95 (0.88‐1.03)

*Note*: Model adjusted for age, sex, BMI, ethnicity, place of residence, education and hot tea consumption.

Abbreviations: CI, confidence interval; HR, hazard ratio (see text for details on adjustment); Q, quintile.

^a^ HR and 95% CI for the increase in one quintile of each variable of meat intake.

^b^ HRs for white meat refer to the model including each type of meat separately. Separate analyses for chicken and fish intake did not provide additional insight (not shown in detail).

**TABLE 4:** Hazard ratios of gastric cancer for quintiles of meat consumption

N cases/non‐cases of gastric cancer	Q1	Q2	Q3	Q4	Q5	
	55/9451	70/9692	85/9907	80/9537	67/9615	
	HR (95% CI)	HR (95% CI)	HR (95% CI)	HR (95% CI)	HR (95% CI)	Continuous^a^
Total red meat	1.00 (Ref)	1.29 (0.90‐1.85)	1.58 (1.11‐2.24)	1.57 (1.10‐2.24)	1.37 (0.94‐1.99)	1.08 (1.00‐1.17)
Red meat	1.00 (Ref)	1.20 (0.83‐1.74)	1.47 (1.02‐2.13)	1.54 (1.06‐2.25)	1.40 (0.95‐2.07)	1.09 (1.00‐1.18)
Organ meat	1.00 (Ref)	0.80 (0.54‐1.19)	0.92 (0.62‐1.37)	0.92 (0.62‐1.36)	0.82 (0.55‐1.22)	0.98 (0.90‐1.06)
Processed meat	1.00 (Ref)	0.90 (0.63‐1.28)	0.60 (0.40‐0.90)	1.20 (0.86‐1.66)	1.19 (0.86‐1.66)	1.03 (0.96‐1.11)
White meat^b^	1.00 (Ref)	0.84 (0.62‐1.16)	0.77 (0.55‐1.07)	0.83 (0.60‐1.16)	0.81 (0.57‐1.13)	0.96 (0.89‐1.04)

*Note*: Model adjusted for age, sex, BMI, ethnicity, place of residence, education and fruit consumption.

Abbreviations: CI, confidence interval; HR, hazard ratio (see text for details on adjustment); Q, quintile.

^a^ HR and 95% CI for the increase in one quintile of each variable of meat intake.

^b^ HRs for white meat refer to the model including each type of meat separately. Separate analyses for chicken and fish intake did not provide additional insight (not shown in detail).

The analysis on meat intake and risk of EC included 340 cases with complete data. Subjects diagnosed with EC were older (mean age 58.8 against 52.0 in the whole cohort), with lower BMI (24.2 against 26.7). A small difference was found according to both total red meat consumption (7.92 g/1000 kcal/day against 7.12 g/1000 kcal/day), and unprocessed red meat (5.99 g/1000 kcal/day against 5.77 g/1000 kcal/day). In the multivariate analysis, EC risk was not associated with any type of meat consumption, although a small excess risk was noticed for high intake of red meat (HR = 1.33, 95% CI = 0.93‐1.93, *P* = .118), corresponding to a 6% higher risk when considering the variable as continuous (95% CI = 0.98‐1.15, *P* = .153). Results based on the substitution method were similar to those reported above (not shown in detail).

The risk of ESCC (n = 289) was not associated with intake of any type of meat. The analysis on esophageal adenocarcinoma was hampered by the small number of deaths (n = 19), with trends showing higher risk associated with red meat intake and lower risk for white meat intake, but not significant.

Stratification by cooking method did not produce noteworthy results (not shown in detail). In the analyses stratified by sex, associations were suggested in women with processed meat (HR dose‐response 0.84, 95% CI = 0.75‐0.95, *P* = .007) and with red meat (HR dose response 1.13, 95% CI = 1.00‐1.27, *P* = .042). The inverse relation for increasing processed meat intake was confirmed among women diagnosed with ESCC (*P* = .006). Details are shown in Table S2.

The analysis on GC included 348 cases with complete data. These subjects were older (mean age 58.6 against 52.0), with a slightly lower BMI (25.5 against 26.7), a mildly higher intake of total red meat (mean 8.55 g/1000 kcal/day against 8.12 g/1000 kcal/day) and red meat (mean 6.35 g/1000 kcal/day against 5.77 g/1000 kcal/day). GC risk was associated with red meat consumption (HR dose response 1.09; 95% CI = 1.00‐1.18 *P* = .043). In particular, higher risk was found in all categories of meat consumption compared to no consumption, with little increment of the excess risk among the categories of consumption above the reference. No associations were found for intake of other meat types. The HR for total red meat was equal to 1.08 (95% CI = 1.00‐1.17, *P* = .049). Results based on substitution method were similar to those reported above (not shown in detail).

When considering the different subsites, cardia GC risk (n = 205) was associated with red meat consumption with a trend similar to that found for GC overall (HR 1.08, 95% CI = 1.00‐1.17), although these results were not formally significant. Risk of non‐cardia GC (n = 92) was also associated with red meat intake (HR dose‐response 1.17, 95% CI = 0.99‐1.37). Stratification by cooking method did not produce further insights in the results (not shown in detail).

In the analysis stratified by sex, the association of GC risk with intake of red meat was stronger in men than in women, particularly for non‐cardia GC (HR dose‐response 1.23, 95% CI = 1.02‐1.48). Stratification by Turkmen ethnicity did not reveal heterogeneity for consumption of unprocessed red meat (*P* heterogeneity = .12), whereas the HR for total red meat consumption was higher in non‐Turkmen than in Turkmen (*P* heterogeneity = .04). Details are reported in Table S2.

When stratifying by opium use (Table 5), unprocessed red meat and total red meat were associated with GC risk within current users, although the results across categories of opium use were not significantly different.

**TABLE 5:** HRs of esophageal cancer and gastric cancer for increase in one quintile of meat intake stratified by opium use

Type of meat	Esophageal cancer				Gastric cancer			
	Never	Former	Current	*P* het.	Never	Former	Current	*P* het.
	280/41 278^a^	7/1058^a^	88/7334^a^		276/41 282^a^	14/1051^a^	83/7339^a^	
Red meat	1.04 0.95‐1.15	0.95 0.51‐1.79	1.15 0.98‐1.37	.6	1.08 0.98‐1.19	0.70 0.43‐1.14	1.22 1.03‐1.45	.1
Processed meat	0.96 0.87‐1.05	1.04 0.59‐1.83	0.98 0.84‐1.15	.4	1.04 0.95‐1.14	0.99 0.67‐1.46	0.99 0.85‐1.15	.5
Organ meat	0.95 0.86‐1.05	1.53 0.78‐2.98	0.96 0.80‐1.14	.4	0.95 0.86‐1.05	1.42 0.88‐2.29	1.02 0.86‐1.22	.6
White meat	0.95 0.87‐1.04	1.08 0.60‐1.93	0.94 0.80‐1.11	.2	0.95 0.87‐1.04	0.51 0.31‐0.84	1.06 0.90‐1.24	.3
Total red meat	1.00 0.92‐1.10	0.91 0.52‐1.59	1.08 0.92‐1.27	.7	1.06 0.96‐1.16	0.78 0.50‐1.21	1.22 1.03‐1.44	.3

*Note*: Each outcome was adjusted for the selected confounders described in Table S1.

Abbreviation: *P* het., *P*‐value of test of heterogeneity across strata of opium use.

^a^ Number of cases and non‐cases.

We estimated that 26.6% of the GC were attributable to total red meat consumption above the first quintile, and 24.4% to unprocessed red meat above the first quintile.

## 4 DISCUSSION

We observed no association between red meat intake and risk of EC, including its subtypes, while an association was suggested for GC overall and in particular non‐cardia GC. No association was found between intake of white meat and risk of either cancer. Most prospective studies that have investigated the possible role of intake of meat, and specifically red meat, in esophageal and gastric carcinogenesis have been conducted in populations with a moderate‐to‐high intake of this type of food. For example, the daily consumption of meat in the United States has been reported to be equal to 26 g for processed meat, 41 g for unprocessed meat and 43 g for poultry.^25^

Ours is one of the first prospective studies conducted in a population with a high risk of these cancers, and a relatively low intake of red meat. To our knowledge the only previous study conducted under comparable circumstances is the analysis of the Linxian Cohort by Tran et al^26^: that population had a very low intake of meat (all types combined), with 14% of cohort members consuming more than 12 servings per year; or about 5 g/day considering an average portion of 150 g. These authors reported an inverse association with EC, that they interpreted as possible result of confounding by socioeconomic status, and no association with either cardia or non‐cardia GC. A second cohort study was conducted in a Chinese population with slightly higher intake of red meat than GCS: the HR of GC for the comparison of intake of red meat above 66.5 g/day vs 36.0 or less g/day was 1.45 (95% CI = 0.93‐2.28).^27^

Our results are overall consistent with those reported from populations characterized by lower incidence of these cancers and higher intake of red meat. For example, in the European EPIC cohort, mean intake of red and processed meat combined in the lowest quartile was 53.4 g/day in men and 39.7 g/day in women.^28^ We observed a positive association between high intake of total red meat and unprocessed red meat, with HR of GC equal to 1.41 in the highest quintile of intake, with mean consumption of 42.8 g/day: a linear extrapolation of our results would result in HR of 2.29 per 100 g/day, comparable to those observed in other large‐scale cohort studies (eg, HR 1.73 per 100 g/day in the EPIC study^28^). The results of a recent pooled analysis of case‐control studies of GC found odds ratios (ORs) for the comparison of high vs low tertile of 1.24 (95% CI = 1.00‐1.53) for red meat, 1.23 (95% CI = 1.06‐1.43) for processed meat and 1.30 (95% CI = 1.09‐1.55) for total meat, with a dose‐response relationship for red and processed meat.^29^

Conversely, no relation was found between white meat and any of the cancers considered, including cancer subtypes. This is unlikely to be a chance result, because of the relatively high intake of this type of food in the GCS, and represents a difference compared to previous studies, that suggested a protective effect of white meat in the order of 20% for both EC and GC.^4^, ^5^ Our result may also be due to residual confounding, where white meat is mostly eaten in GCS by people of low socioeconomic status, in contrast to more health‐conscious people in other populations, for example, in Europe.^29^

Possible mechanisms of esophageal and gastric carcinogenesis of red meat include the endogenous formation of genotoxic *N*‐nitroso compounds (NOCs), heterocyclic amines and polycyclic aromatic hydrocarbons produced through high‐temperature cooking, as well as iron and agents associated with meat processing, that may cause oxidative stress. Meat intake has been also correlated to promotion of the growth of Hp,^30^ which is the main risk factor for non‐cardia GC.

We found no association between EC and GC and processed or organ meat. While for the latter results are lacking, intake of processed meat has been widely associated with both cancers.^1^, ^31^ Possible explanations of our results on processed meat are the low intake in our population, and the different types of processed meat consumed in Iran (primarily beef‐based) compared to countries where an association has been detected (primarily pork‐based).

The exploratory analysis based on stratification by sex suggested some minor differences between men and women, with an apparently stronger association between red meat intake and risk of non‐cardia GC among men, and a negative association between processed meat intake and EC risk among women. Also, an apparent stronger effect of total red meat consumption was suggested among non‐Turkmen compared to Turkmen, which may reflect differences in the pattern of consumption that were not captured in our FFQ. These results should be interpreted with caution because of multiple comparisons and low consumption of processed meat, but might also reflect unmeasured patterns of consumption, effect modifiers or confounders.

In addition, stratification by opium use did not show heterogeneity in the effect of unprocessed red meat intake across strata of opium in the risk of GC, suggesting an independent effect of the two risk factors.

When considering the different methods of meat preparation, we observed no apparent effect of any methods on the results. The data available for this analysis, however, were rather crude, and there was substantial overlap between the different methods, hampering the ability to identify the role played by each of them.

The main strength of the present study is the prospective design. Our study population is characterized by large sample size and number of cancer cases compared to other cohort studies, providing the possibility to describe associations for different subtypes of cancers with robust results. In particular, GCS offers the unique opportunity to investigate cardia GC, as far as it represents a high‐risk population for this less common type of cancer. Also, we used detailed and validated questionnaire to collect information describing the study population and we could therefore adjust for different potential confounders, including opium use, salt intake and hot tea consumption.

This is the first large cohort study from a high‐risk population to explore the association between multiple types of meat and both EC and GC, including their subtypes. The majority of white meat consumed by GCS members consisted of chicken, and we did not find a relationship between different types of white meat and cancer. In addition, we did analyze the role of organ meat consumption, which to our knowledge has not been investigated in association to either EC or GC before. This is one of the few studies to address the potential effect exerted by type of cooking. Also, this is the first study to investigate the interaction between increasing consumption of red meat and opium use toward risk of GC.

We addressed the outcomes using both unadjusted consumption (g/day) and density‐adjusted consumption (g/1000 kcal/day), offering also the possibility to evidence any difference between the two. This strategy gives further insight to the results for unadjusted intake, as far as it is corrected for the total daily energy intake of subjects which may indicate an unbalanced diet.

Our study has some limitations. Our analysis lacks adjustment for Hp infection, which in this population plays a key role on both cardia and non‐cardia GC.^32^ On the other hand, as infection rate nears 100% in Iran, no stratification would have been possible. Furthermore, due to the small number of events, we could not describe any robust result on EAC, which in this population is less frequent than ESCC.

In addition, we could not separate in the analysis the different types of red meat, for example, beef, mutton, goat and camel or different organs, for example, liver, kidney and heart. According to the Food and Agriculture Organization's Food Balance Sheet, production of beef meat in Iran in 2018 accounted for 463 000 tons, that of mutton and goat meat to 414 000 tons and that of other types of red meat to 10 000 tons.^33^ A more detailed analysis based on specific types of red meat might highlight possible heterogeneity in the association with EC and GC, that has been evidenced in the analysis considering them together. Finally, information on meat preservation was not available, although in preliminary analyses we adjusted the results for salt intake, and found no evidence of confounding.

In conclusion, our study shows a 9% and 8% increased risk of GC for an increase in one quintile of the distribution of red meat and total red meat intake, respectively. If causal, this effect would correspond to approximately one fourth of cases of GC attributable to red meat consumption above the bottom quintile. This is comparable to figures that can be derived from other populations (eg, in the EPIC cohort,^29^ 22.2% of GC are attributable to red meat consumption above the bottom quartile). No associations were found between intake of other types of meat and GC, as well as between intake of any type of meat and EC. Further prospective studies from high‐risk populations, including stratified analyses according to cancer subsites, as well as for different types of red, processed, organ and white meat would complement our findings with the overall goal of better characterizing the etiologic role of this type of food on these cancers.

## AUTHOR CONTRIBUTIONS

Conception and design of study: Giulia Collatuzzo, Arash Etemadi, Sanford M. Dawsey, Christian C. Abnet, Farin Kamangar, Paul Brennan, Paolo Boffetta, Reza Malekzadeh; acquisition of data: Arash Etemadi, Masoud Sotoudeh, Arash Nikmanesh, Hossein Poustchi, Masoud Khoshnia, Akram Pourshams, Maryam Hashemian, Gholamreza Roshandel, Reza Malekzadeh; analysis and interpretation of data: Giulia Collatuzzo, Arash Etemadi, Paolo Boffetta; drafting the article: Giulia Collatuzzo, Arash Etemadi, Paolo Boffetta; revising the article critically for important intellectual content: Sanford M. Dawsey, Christian C. Abnet, Farin Kamangar, Reza Malekzadeh; approval of final article: all authors. The work reported in the article has been performed by the authors, unless clearly specified in the text.

## CONFLICT OF INTEREST

The authors declare no conflicts of interest.

## ETHICS STATEMENT

Our study was approved by the institutional review boards of the Digestive Disease Research Center of Tehran University of Medical Sciences, the US National Cancer Institute, the World Health Organization's International Agency for Research on Cancer. All participants provided written informed consent before enrollment.

## Supporting information


**Appendix S1** Supporting Information.

## Data Availability

The data that support the findings of our study are available from the corresponding author upon reasonable request and approval of the Steering Committee of the Golestan Cohort Study.
