# Supplementary material for: Meat consumption and risk of esophageal and gastric cancer in the Golestan Cohort Study, Iran
Source: Int J Cancer. 2022 May 17;151(7):1005–12. doi: 10.1002/ijc.34056 (PMC9543688; doi:10.1002/ijc.34056)

Supplementary Material for:

Meat consumption and risk of esophageal and gastric cancer in the Golestan Cohort Study, Iran

Giulia Collatuzzo, Arash Etemadi, Masoud Sotoudeh, Arash Nikmanesh, Hossein Poustchi, Masoud Khoshnia, Akram Pourshams, Maryam Hashemian, Gholamreza Roshandel, Sanford M. Dawsey, Christian C. Abnet, Farin Kamangar, Paul Brennan, Paolo Boffetta, Reza Malekzadeh

Table of contents:

Supplementary Table 1: Variables included for adjustments in regression models for specific outcomes

Supplementary Table 2: Hazard ratios for types of meat consumption, stratified by sex

Supplementary Figure 1: Hazard ratio for increase in one quintile of meat intake by subtype of esophageal and gastric cancer

Supplementary Table 1: Variables included for adjustments in regression models for specific outcomes.

Outcome	Specific adjustments
EC	Education, tea temperature
ESCC	Education, tea temperature, fruit, tobacco smoking, salt intake
EAC	Education, fruit, vegetables, tobacco smoking, opium use
GC	Education, fruit
Cardia GC	Education, tea temperature, fruit
Non-cardia GC	Education, tea temperature, fruit, vegetables, opium use

Notes: The a-priori adjustments include sex, age, BMI, urban/rural residence, Turkmen ethnicity
Additional models also included (i) unprocessed red meat, processed meat, organ meat and white meat; (ii) total red meat and white meat
Substitution models also included variables for each single meat type and for total red meat
EC, esophageal cancer; GC, gastric cancer; BMI, Body Mass Index

Supplementary Table 2: Hazard ratios for types of meat consumption, stratified by sex

**Type of meat**	**GC**		**CGC**		**NCGC**		**EC**		**ESCC**		**EAC**	
	F	M	F	M	F	M	F	M	F	M	F	M
Red meat	1.06, 0.90-1.24	1.10, 1.00-1.21	1.13, 0.92-1.39	1.07, 0.94-1.21	0.99, 0.70-1.39	1.23, 1.02-1.48	1.13, 1.00-1.27	0.99, 0.88-1.11	1.11, 0.97-1.27	1.00, 0.89-1.14	1.13, 0.68-1.87	1.25, 0.77-2.05
Processed meat	0.97, 0.83-1.13	1.05, 0.97-1.15	0.90, 0.73-1.10	1.07, 0.96-1.20	1.11, 0.82-1.49	1.00, 0.85-1.18	0.84, 0.76-0.95	1.09, 0.98-1.21	0.83, 0.72-0.95	1.09, 0.97-1.22	1.30, 0.85-1.99	0.96, 0.60-1.55
Organ meat	0.91, 0.78-1.07	1.01, 0.92-1.12	0.97, 0.79-1.20	1.00, 0.88-1.13	0.81, 0.58-1.11	1.11, 0.91-1.34	1.03, 0.91-1.16	0.90, 0.79-1.01	1.04, 0.91-1.18	0.89, 0.79-1.02	0.84, 0.51-1.38	0.87, 0.52-1.45
White meat	1.02, 0.88-1.17	0.94, 0.85-1.03	0.96, 0.80-1.15	0.95, 0.84-1.07	1.27, 0.92-1.77	0.86, 0.72-1.03	0.97, 0.87-1.08	0.95, 0.85-1.06	1.01, 0.89-1.14	0.94, 0.84-1.06	0.88, 0.56-1.41	0.69, 0.43-1.13
Total red meat	1.05, 0.91-1.22	1.09, 1.00-1.20	1.08, 0.89-1.32	1.10, 0.98-1.24	1.09, 0.80-1.50	1.15, 0.97-1.38	1.04, 0.93-1.17	0.99, 0.89-1.10	1.02, 0.90-1.15	1.01, 0.90-1.14	1.45, 0.89-2.46	1.15, 0.72-1.83

HR are adjusted for the selected confounders as described in Supplementary Table 1; they indicate the risk for an increase of one quintile of meat intake

GC, gastric cancer; CGC, cardia gastric cancer; NCGC, non-cardia gastric cancer; EC, esophageal cancer; ESCC, esophageal squamous cell carcinoma; EAC, esophageal adenocarcinoma

Supplementary figure 1. Hazard ratio for increase in one quintile of meat intake by subtype of esophageal and gastric cancer.

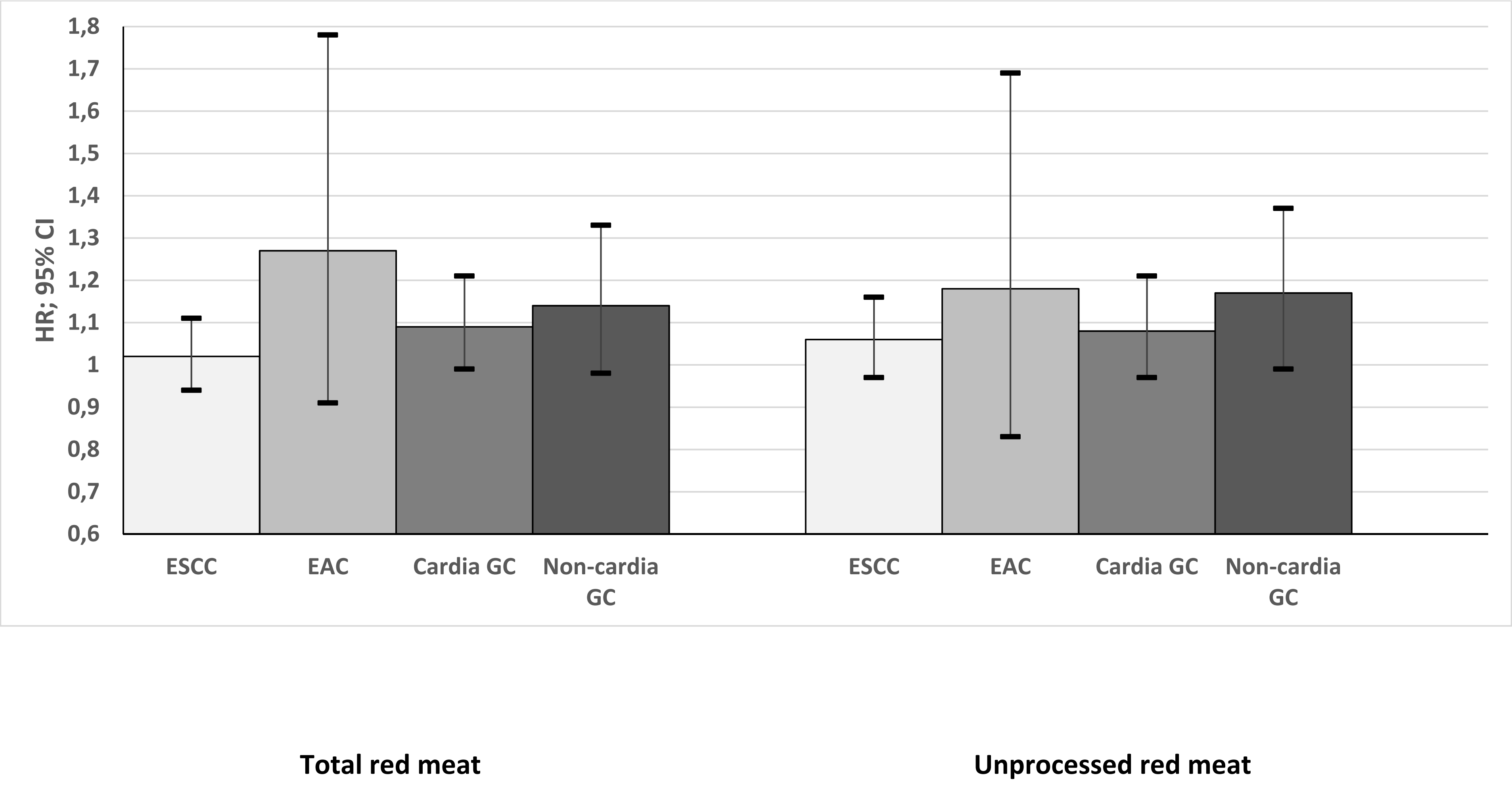

Supplement: Supplementary file 1 — Appendix S1 Supporting Information. [file IJC-151-1005-s001.pdf]
